# *XadA*-like adhesin XADA2 regulates biofilm formation in *X. fastidiosa* subsp. *fastidiosa* putatively by engaging oleic-acid derived oxylipins

**DOI:** 10.1007/s11033-025-10259-y

**Published:** 2025-02-25

**Authors:** Valeria Scala, Manuel Salustri, Marcus Vinicius Merfa, Marzia Beccaccioli, Leonardo Lascala, Leonardo De La Fuente, Massimo Reverberi

**Affiliations:** 1 Council for Agricultural Research and Economics, Research Centre for Plant Protection and Certification of Rome, 00156 Rome, Italy; 2 Department of Environmental Biology, Sapienza University of Rome, Rome, Italy; 3 Department of Plant Pathology, Infectious Diseases Institute, College of Food, Agricultural, and Environmental Sciences, Columbus, US; 4 Department of Entomology and Plant Pathology, Auburn University, Auburn, OH US

**Keywords:** Oxylipins, Adhesion, Biofilm, Xylella fastidiosa, ODS

## Abstract

**Background:**

The oxylipins 10-HpOME and 7,10-DiHoME derive from oleic acid and have been extensively studied for their ability to regulate contractions, microcolony formation and biofilm formation in the model organism *Pseudomonas aeruginosa*.

**Methods and results:**

*Xylella fastidiosa* subsp. *pauca* strain de Donno has been reported to produce 10-HpOME and 7,10-DiHOME in vivo when inoculated in the model plant *Nicotiana tabacum* or in naturally occurring infected olive trees. In this study, we deciphered the relationship among cell adhesion and oxylipins in *Xylella fastidiosa* subsp. *fastidiosa* (Temecula1 strain) and subsp. *multiplex* (AlmaEM3 strain). The role of the PD0744 gene, encoding for XadA2, a non-fimbrial adhesin belonging to the trimeric autotransporter family, probably involved in the surface attachment required in the initial phase of biofilm formation was investigated. *PD0744* deletion mutants in two *X. fastidiosa* strains were generated, through homologous recombination, and the impact of its deletion on bacterial lifestyle was assessed. In vitro assays were performed to characterize the mutant phenotype, particularly in twitching motility and its capability to grow and form biofilm. Mutants showed a reduced twitching motility and biofilm formation compared to wild type strains. HPLC–MS/MS analysis revealed a decrease in 7,10-DiHOME production together with an increase of its precursor 10-HpOME in the mutants.

**Conclusions:**

7,10-DiHOME could be a crucial signaling molecule to promote biofilm formation and twitching motility, whose synthesis likely depends on a signal transduction requiring the presence of the adhesin XadA2 and thus not working if this protein is depleted. These results help understanding the complex regulation of biofilm formation in this devastating pathogen.

**Supplementary Information:**

The online version contains supplementary material available at 10.1007/s11033-025-10259-y.

## Introduction

*X. fastidiosa* infects a wide range of plant species with or without the manifestation of clear symptoms, often living as an endophyte within host plants [1]. It is believed that symptoms occur when the pathogen multiplies inside the xylem vessels, switching from a planktonic to a sessile lifestyle, forming cell aggregates (biofilm) that block the sap flow [2]. Proteins involved in this process are commonly known as adhesins, being distinguished as fimbrial and non-fimbrial [3]. Fimbrial adhesins FimA and FimF constitute Type I short pili, involved in cell–cell adhesion and biofilm formation, while fimbrial adhesins Pil are the building blocks of long Type IV pili, required for a cell motility named twitching [4]. This type of motility, consisting in a translocation that involves intermittent extension, anchoring and retraction of the pili, allows *X. fastidiosa* to migrate along the xylem even against the sap stream, i.e., in the basipetal direction, and to colonise new xylem vessels [5]. Knockout mutants of *X. fastidiosa* for fimbrial adhesins involved in building Type IV pili, as pilB and pilQ, have an impairment in twitching motility and in basipetal movement *in planta*, while showing an increase in biofilm formation [6]. On the other hand, knockout mutants for adhesins constituting Type I pilus, precisely FimA, exhibited increased twitching motility, being instead unable to form biofilm in vitro [6]*.* Such results demonstrate antagonistic effects of Type IV and Type I pili on twitching motility and biofilm formation, suggesting that *X. fastidiosa* should balance, depending on the stage of its colonisation within the plant xylem or the insect foregut, the synthesis of long and short pili [6, 7].

*X. fastidiosa* also possesses non-fimbrial adhesins, i.e., outer membrane proteins mainly contributing to the adhesion of cells to surfaces. These type of adhesins are not components of pili. Among non-fimbrial adhesins there are several hemagglutinins [8].The xadA-like adhesins (XadA1, XadA2, XadA3) belong to the trimeric autotransporter adhesin (TAA) family [9].

*hxfA* or *hxfB* (hemagglutinins) knockout mutants of *X. fastidiosa* subsp. *fastidiosa* strain Temecula1 result in hypervirulent strains when inoculated into grapevine stems and in a loss of microcolony and biofilm formation in vitro and *in planta* [10]. These strains bind less effectively to polysaccharides in the foregut of the insect vector and are therefore less transmissible [10].

The trimeric autotransporter adhesin harboured by *X. fastidiosa* are homologous to the *Xanthomonas oryzae* protein XadA (*Xanthomonas* adhesin-like protein A), which shares similarity with several non fimbrial adhesins of animal pathogenic bacteria, such as YadA of *Yersinia enterocolitica.* YadA mediates adhesion to extracellular matrix components of eukaryotic host cells and isa decisive virulence factor [11]. XadA, an outer membrane protein, is required for full virulence and normal colony morphology of *X. oryzae* pv. *oryzae* [12]. The xadA-homolog trimeric autotransporters found in *X. fastidiosa* are XadA1, XadA2, and XadA3. They are the top abundant proteins identified in the outer membrane vesicle (OMV) of *X. fastidiosa* subsp. *fastidiosa* strains Temecula1 and subsp. *pauca* strains 9a5c and Fb7 [9].

In *X. fastidiosa* subsp. *pauca* strain 9a5c the non-fimbrial protein XadA1 was detected in all phases of biofilm development, and XadA2 was detected mainly in later phases of biofilm development [13]. In the Temecula1 strain, XadA1 mutants were reported to be defective in adhesion as single cells to glass surfaces, but cell–cell aggregates could still form. Thus, XadA1 is apparently involved in the initial adhesion of Temecula1 cells [3]. The other non-fimbrial protein evaluated in 9a5c strain, XadA2, was observed mainly only after 10 days. Nevertheless, recent studies in 9a5c strain highlighted the low efficiency of XadA2 in increasing the cell–cell adhesion and thus the biofilm expansion, while having a crucial role in surface attachment, because of the great affinity both to insect chitin and xylem cellulose [14].

Overall, the virulence of X. *fastidiosa* appears to be a balance between its migratory capacity, thus the colonisation of new vessels, and the ability to form extended biofilms [15]. This complex phenomenon needs to be fine-tuned by regulatory signalling: So far, mechanisms of the Diffusible Signalling Factor (DSF)—based *quorum sensing* model has been extensively described [16, 17]. DSFs are monounsaturated fatty acids, produced by the activity of the DSF synthase RpfF [18]. Molecules that act like DSFs are generically defined as autoinducers. The achievement of a specific concentration of autoinducers, following the increase in the bacterial population, triggers the expression of genes involved in cell aggregation, in adhesion to surfaces and in biofilm formation, such as *fimA*, encoding for a fimbrial adhesin, and hxfA and hxfB, encoding for hemagglutinins. Conversely, it reduces the expression of genes implicated in cell motility and in plant cell wall degradation [16].

Recent studies highlighted ODS (oxylipin-dependent quorum sensing system) regulated by oxylipins, derived from oleic acid, in *Pseudomonas aeruginosa* [19]. Oxylipins are common cell signalling molecules among eukaryotes [20], however, little is known about their role in bacterial physiology. Oxylipin production is catalyzed by lipoxygenase (LOX) and dioxygenase (DOX), though it can also result from the chemical (non-enzymatic) oxidation of fatty acids due to reactive oxygen (ROS) and nitrogen species (NOS) [21]. Further studies have shown that *P. aeruginosa* has several dioxygenases including a diol synthase that catalyses the stereospecific oxygenation of oleic acid [21]. When oleic acid is used as a substrate, the enzyme diol synthase can synthesise 10(S)-hydroxy-8(E)octadecenoic acid (10-HOME) and 7S,10 Dihydroxy-8(E)octadecenoic acid (7,10-DiHOME), involved in various biological processes, including motility, biofilm formation and virulence [22]. More precisely, in *P. aeruginosa* these oxylipins inhibit active flagellum-driven type motility while stimulating the expression of type IV pili, thus promoting twitching motility. Consequently, these compounds promote the aggregation of the bacterium in micro-colonies and the formation of biofilm in vitro, but also in vivo in *Drosophila melanogaster* [21]. 10-HOME and 7,10-DiHOME were produced by the plant pathogen *X*. *fastidiosa* subsp. *pauca* strain de Donno both in vitro and in vivo when inoculated in the model plant *Nicotiana tabacum* [23], as well as in olive trees infected by *X*. *fastidiosa* subsp. *pauca* [24]. These observations suggest that the production of oxylipins and their role in bacterial cell communication is not limited to *P. aeruginosa* but is common to multiple bacterial families [19]. In this study, we aimed at confirming the production of 10-HOME and 7,10-DiHOME in Temecula1 and in AlmaEM3 strains, both in cells and in culture filtrate, and to correlate them with the deletion of the XadA2 adhesin. This protein is likely involved in the surface attachment required for biofilm formation and whose deletion could thus likely perturb the whole signalling system leading to successive biofilm formation phases, in which the intercellular adhesion is the main process.

## Materials and methods

### Bacterial strains, media, and culture conditions

*X. fastidiosa* subsp. *fastidiosa* strain Temecula1 and subsp. *multiplex* strain AlmaEM3, isolated from infected grapevine in California and blueberry in Georgia, respectively, were used in this study. Strains were cultured in PW agar plates, modified by omitting phenol red and using 1.8 g L^−1^ of bovine serum albumin (BSA; Gibco Life Sciences Technology), for 1 week at 28 °C from − 80 °C glycerol stock, re-streaked onto new PW plates, and cultured for another week before use [7]. PD3 medium was used for culturing and suspending cells in liquid. Whenever needed, antibiotics kanamycin (Km) and chloramphenicol (Cm) were used at concentrations of 30 and 10 g mL^−1^, respectively.

### DNA extraction

DNA was extracted with the Quick-DNA fungal/bacterial miniprep kit (Zymo Research) using the manufacturer’s protocol. Cells cultured on PW agar plates were resuspended in sterile Milli-Q water and stored at − 20 °C until use. DNA was extracted by disrupting cells for 5 min using a Mini-BeadBeater-96 (BioSpec Products).

### Knockout construction

Mutation of PD0744 was performed as previously described [25]. In summary, the upstream (810 bp) and downstream (805 bp) regions flanking this gene of interest were amplified from the X. *fastidiosa* strain Temecula1 genome using the corresponding Upstream_Forward/Upstream_Reverse and Downstream_Forward/Downstream_Reverse pairs of primers, respectively (Table 1). These primers contain overlapping nucleotides with the Km resistance cassette used in this study to allow for extension-overlap PCR to fuse all three fragments together. The Km resistance cassette was amplified from the pUC4K plasmid [26] using the Kanamycin_Forward and Kanamycin_Reverse pair of primers (Table 1). Each of these three fragments (upstream and downstream regions, and Km resistance cassette) were then fused together via overlap-extension PCR [25] to obtain the targeting construct to delete PD0744 via homologous recombination. For the overlap-extension PCR, the three fragments were purified from agarose gel, mixed in equal proportions, and amplified using the Upstream_Forward and Downstream_Reverse primers.

**Table 1:** List of primer pairs used for gene knockout and deletion confirmation

Amplicon name	Primer name	Primer Sequence (5′-3′)	Amplicon Length (bp)
Kanamycin resistance cassette from pUC4K plasmid	Kanamycin_Forward	GTCTGCCTCGTGAAG	1204
	Kanamycin_Reverse	AAGCCACGTTGTGT	
Upstream flanking region	Upstream_Forward:	ACCGATACAGTGGCATTAGG	810
	Upstream_Reverse:	CTTCACGAGGCAGACGCTACGTTTCACCGACCCTTCGTTT	
Downstream flanking region	Downstream_Forward:	ACACAACGTGGCTTCCTCAGTGTGCTGTGTGTAA	905
	Downstream_Reverse:	CCGAGGATGTCTCGCAAT	
Fusion construct for mutagenesis	Upstream_Forward:	ACCGATACAGTGGCATTAGG	2919
	Downstream_Reverse:	CCGAGGATGTCTCGCAAT	
Internal control (within gene of interest)	Int_Forward	TGCTGAATTCTAAGTCG	1400
	Int_Reverse	ATTGACCGCATCCGTATC	

The fusion product (2919 bp), which contained the Km resistance cassette flanked by the upstream and downstream regions of the target (PD0744), was purified from gel and stored at − 20 °C before use. PCR reactions were performed with a standard protocol, using an iProof High-Fidelity PCR kit (Bio-Rad) in an S1000 thermal cycler (Bio-Rad). PCR products were gel-purified using a 5PRIME gel extract minikit (Bio-Rad) and used for the transformation described below (Figure S1).

### Natural transformation of X. fastidiosa strains with PCR template and confirmation of mutants

Transformation of *X. fastidiosa* cells was performed with the gel-purified templates, using a natural transformation protocol as previously described [25]. Briefly, recipient *X. fastidiosa* strain Temecula1 or AlmaEM3 cultures from PW plates were suspended in PD3 liquid medium and optical density at 600 nm (OD600) was adjusted to 0.25. 10 µl of this suspension was spotted onto PD3 agar plates, 10 µl of PCR template was added on top of the spots, and the spots were dried before incubation at 28 °C for 3 days. After 3 days, spots were suspended in 0.5 ml of PD3. Aliquots were spread plated onto PW plates containing necessary antibiotics for selection. After 2 weeks of incubation at 28 °C, mutant CFU were restreaked onto new antibiotic PW plates. Ten colonies per strain were restreaked onto new antibiotic plates. After confirming that the colonies were resistant to the corresponding antibiotic, one colony per strain was selected for further analysis. Confirmation of gene deletion was performed by PCR. PCR was performed with two sets of primer pairs, namely, the int pairs (int_Forward/int_Reverse) that target a genomic region inside the gene of interest, and the antibiotic primers (Kanamycin_Forward/Kanamycin_Reverse). For each PCR, wild-type strains were included as controls.

### Planktonic growth, biofilm formation and twitching motility

Planktonic growth and Biofilm formation of the mutant and wild-type strains was assessed in 96-well plates after 5 days of growth, as previously described [27]. Briefly, cells were suspended in PD2 broth (OD_600_ 0.8) and 10 µL was inoculated into 96-well plates containing 200 µL of PD2 broth per well. After 5 days of growth, 150 µL of each culture supernatant were transferred to a new 96-well plate to determine the planktonic growth by measuring the OD_600_ of this suspension. Concurrently, biofilm formation was measured by rinsing three times the wells of the original 96-well plate with Milli-Q water and staining cells with 230 µL of a 0.1% crystal violet solution. Wells were then gently rinsed three times with Milli-Q water and destained using 230 µL of 95% ethanol under agitation (150 rpm) for 5 min. The OD_600_ of wells was finally measured to determine biofilm formation. Experiments were repeated three times independently. For each wild-type strain or mutant background eight wells (repetitions) on each plate were used. Twitching motility assessment of wild-type and mutant strains was performed in BSA-free PW plates. Twitching motility was observed for at least 10 transformant colonies. Selected colonies were restreaked, and repeated observation of these colonies for twitching motility was performed [24]. All experiments had at least three biological replicates, unless otherwise stated.

### Lipid analysis

The solvents used for the HPLC / MS phases and for the extraction of the samples are acetonitrile (ACN), isopropanol (iPrOH), methanol (MeOH) and ethyl acetate (EtOAc) purchased from Merck (Darmstadt, Germany);, the formic acid (HCOOH) which was purchased by Fluka (Buchs SG, Swiss). The lipid extraction was carried out on wild type cells of *Xylella fastidiosa*, as well as on their mutants deleted in the PD0744 gene. Cells were grown in PD3 medium for 5 and 8 days, which is the time interval in which Temecula1 and AlmaEM3 produce biofilm in liquid culture [24]. Cells were separated from culture filtrate by centrifugation, and then cells were lyophilized. The internal standards added in each sample was the oxylipin 9-hydroxy-10E, 12Z octadecadienoic acid (9-HODEd4, MW 300.5 g / mol) at final concentration of 2 µM for all samples. For each sample 20 mg of previously lyophilized matrix was taken. Subsequently the matrix was vortexed for about 5 min with 2 mL of extractive solution (iPrOH: H2O: EtOAc 1: 1: 3 v / v, with 0.0025% w / v of Butylated hydroxytoluene (BHT) to prevent peroxidation) and centrifuged for 10 min. After the centrifugation, the upper ethyl acetate phase was transferred to a new tube and dried under a flow of gaseous nitrogen. The pellet was resuspended with the addition of 1.2 mL of EtOAc, then vortexed and centrifuged again. Finally, the upper phase was recovered and dried again under a nitrogen flow. The dried samples were resuspended in 100 μL of MeOH. The HPLC/MS–MS analysis of the lipids were obtained by 1200 series HPLC (Agilent Technologies, Santa Clara, CA, USA) coupled to the triple quadrupole G6420A (Agilent Technologies, Santa Clara, CA, USA) with electrospray ionisation source (ESI). For the analysis of the oxylipins the chromatographic separation was carried out with a C18 column (Zorbax ECLIPSE XDB-C18 rapid resolution HT 4.6 × 50 mm 1.8 μm (Agilent Technologies, Santa Clara, CA, USA). The binary mobile phase consisted of: A (water / ACN 97: 3 v / v containing 0.1% HCOOH), and B (ACN / iPrOH 90:10 v / v). The elution program and the flow rate were the same as those already reported [24]. The column was thermostated at 60 °C. The injection volume was 10 μL. The temperature and flow of the nebulizing and desolvating gas (nitrogen) were 350 °C and 9 L / min, respectively, the atomizing pressure 20 psi. MRM analysis is carried out using the method described in Scala et al., [28] and particularly devoted to quantifying the amount of 10-HpOME and 7,10-diHOME using 9-HODEd4 as internal standard (ISTD).

### Statistical analysis

The results of the HPLC–MS/MS analysis were subjected to normalisation and statistical analysis. The areas of the chromatographic peaks, obtained following the MRM experiments, were normalised to the values of the internal standard. For each set of value, the comparison between the mutants and their respective WT regarding oxylipin levels, total growth, planktonic growth, biofilm formation and fringe width was performed using One-way ANOVA followed by Tukey’ post hoc test performed using GraphPad Prism version 10.0.0 for Windows, GraphPad Software, Boston, Massachusetts USA, www.graphpad.com.

## Results

### Assessment of biofilm formation, planktonic growth and twitching motility assay

**Fig. 1:**
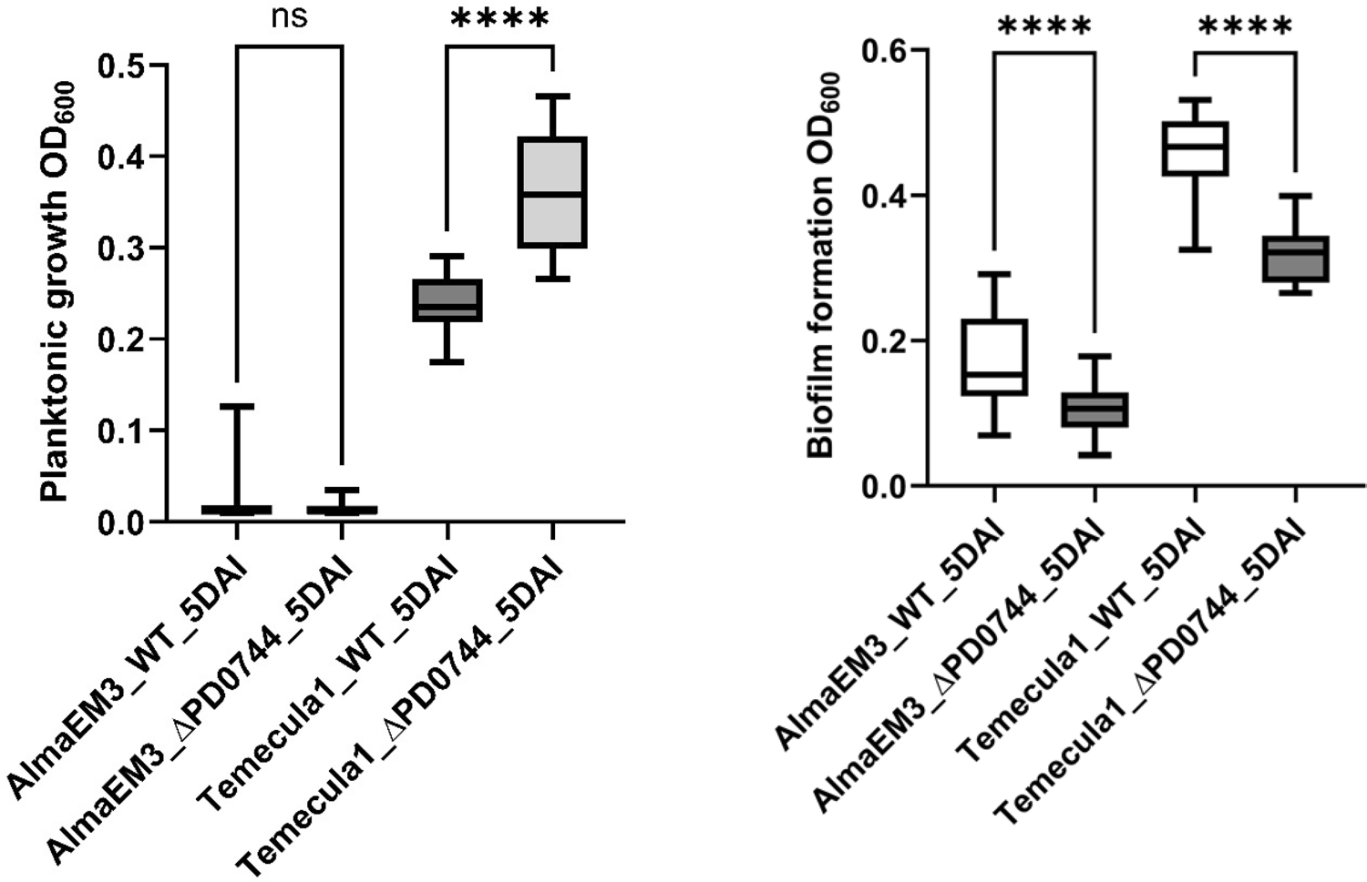
Planktonic growth and biofilm formation by wild type (WT) and PD0744 mutant (*ΔPD0744*) of *X. fastidiosa* subsp. *fastidiosa* strain Temecula1 and subsp. *multiplex* strain AlmaEm3 5 days after inoculation (DAI). Y-axis: Optical density. X-axis: Examined strains. 24 repetitions for each sample for experiment (3 experiments). Boxplots were plotted using Tukey method, values were compared by an ordinary one-way ANOVA followed by the Tukey’ post hoc test. Asterisks indicate p value: **** p < 0.0001; ***p < 0.001; **p < 0.01; *p < 0.05)

**Fig. 2:**
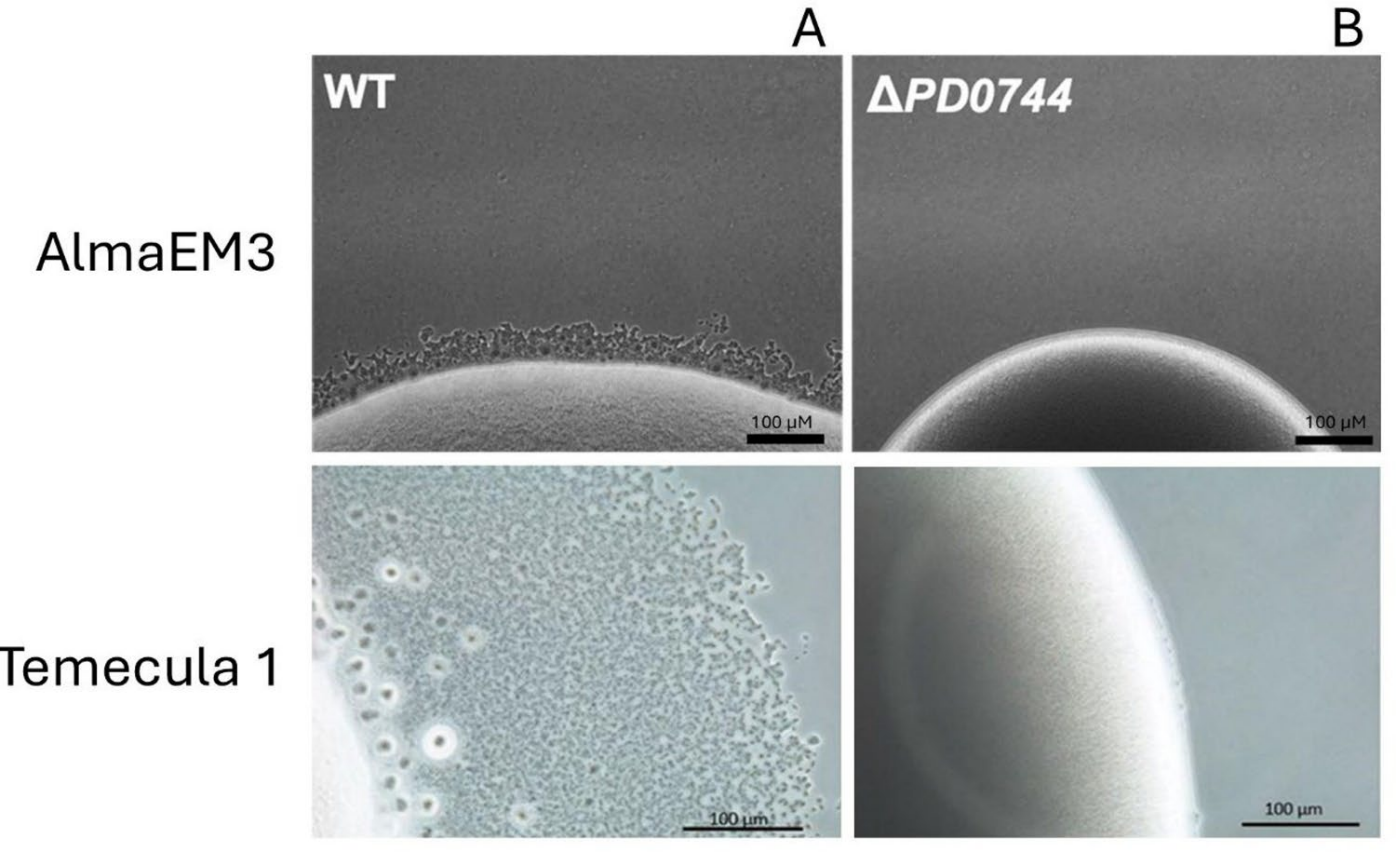
Twitching motility assessment of wild type (WT) and PD0744 mutant (*ΔPD0744*) of *X. fastidiosa* subsp. *fastidiosa* strain Temecula1 and subsp. *multiplex* strain AlmaEm3. The wild-type phenotype **A**. The mutants’ phenotype, in which it is possible to notice the absence of fringes from the inoculum **B**. Images captured with 40 × magnification with Nikon Ti inverted phase contrast inverted microscope. The fringes were measured with NIS-Elements Advanced Research 3.01 (Nikon) software

**Fig. 3:**
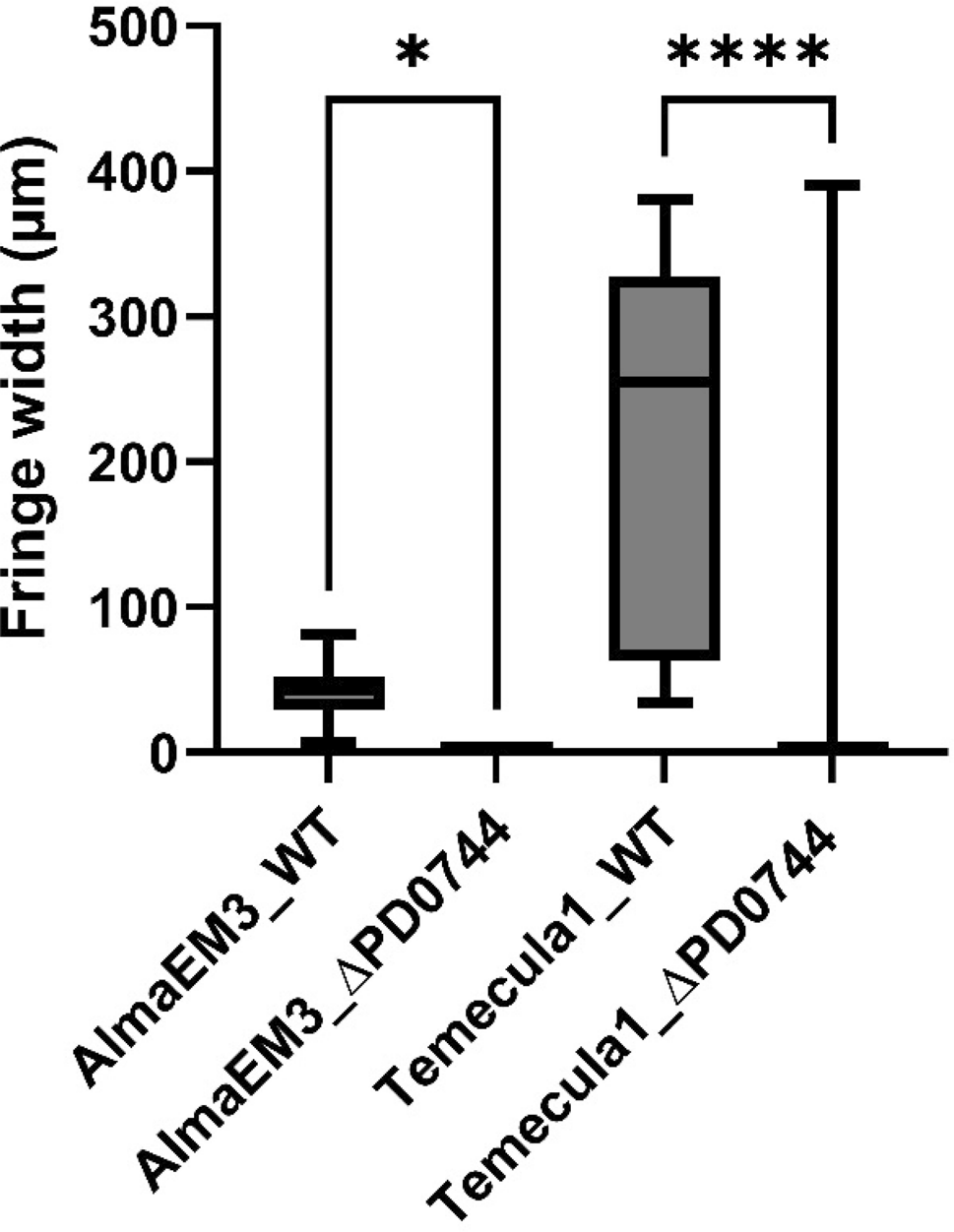
Boxplots showing different values of fringe width length obtained in wild type (WT) and PD0744 mutant of *X. fastidiosa* subsp. *fastidiosa* strain Temecula1 and subsp. *multiplex* strain AlmaEm3. The mean of both distributions is represented by a cross. Error bars represent the standard deviations from the mean. Boxplots were plotted using Tukey method, values were compared by an ordinary one-way ANOVA followed by the Tukey’ post hoc test. Asterisks indicate p value: **** p < 0.0001; ***p < 0.001; **p < 0.01; *p < 0.05)

Wild type and mutant strains were evaluated for growth phenotypes to observe how deletion of *xadA2* (PD0744) affects biofilm formation, planktonic growth and twitching motility. Remarkably, planktonic growth and biofilm development, which were assessed in 96-well microplates, showed significant differences between the examined strains. Biofilm formation significantly decreased (p—value < 0.001) in the mutant strains in comparison to that of their respective wild-type (Fig. 1). The opposite tendency was observed in planktonic growth for Temecula1, with the mutant having significantly (p—value < 0.001) higher planktonic growth than that of the wild-type strain (Fig. 1). In summary, biofilm formation seems to be disrupted in the mutant, likely due to the loss of XadA2 adhesive properties. In AlmaEM3, deletion of *PD0744* did not significantly affect planktonic growth (Fig. 1). The twitching motility was assessed in BSA-free PW plates, as previous studies demonstrated BSA significantly inhibits twitching [29]. Results showed that the mutants have lost motility, as no fringe was found outside the margin of the inoculum (Fig. 2B) in comparison to the wild type strains (Fig. 2A). ∆PD0744 was non-motile, while *X. fastidiosa* Temecula1 and AlmaEM3 WT had a fringe width of 247.4 and 47.02 µm, respectively (mean ± standard error; shown in chart and figure below; Fig. 3).

### Quantification of 10-HpOME and 7,10-diHOME oxylipins

**Fig. 4:**
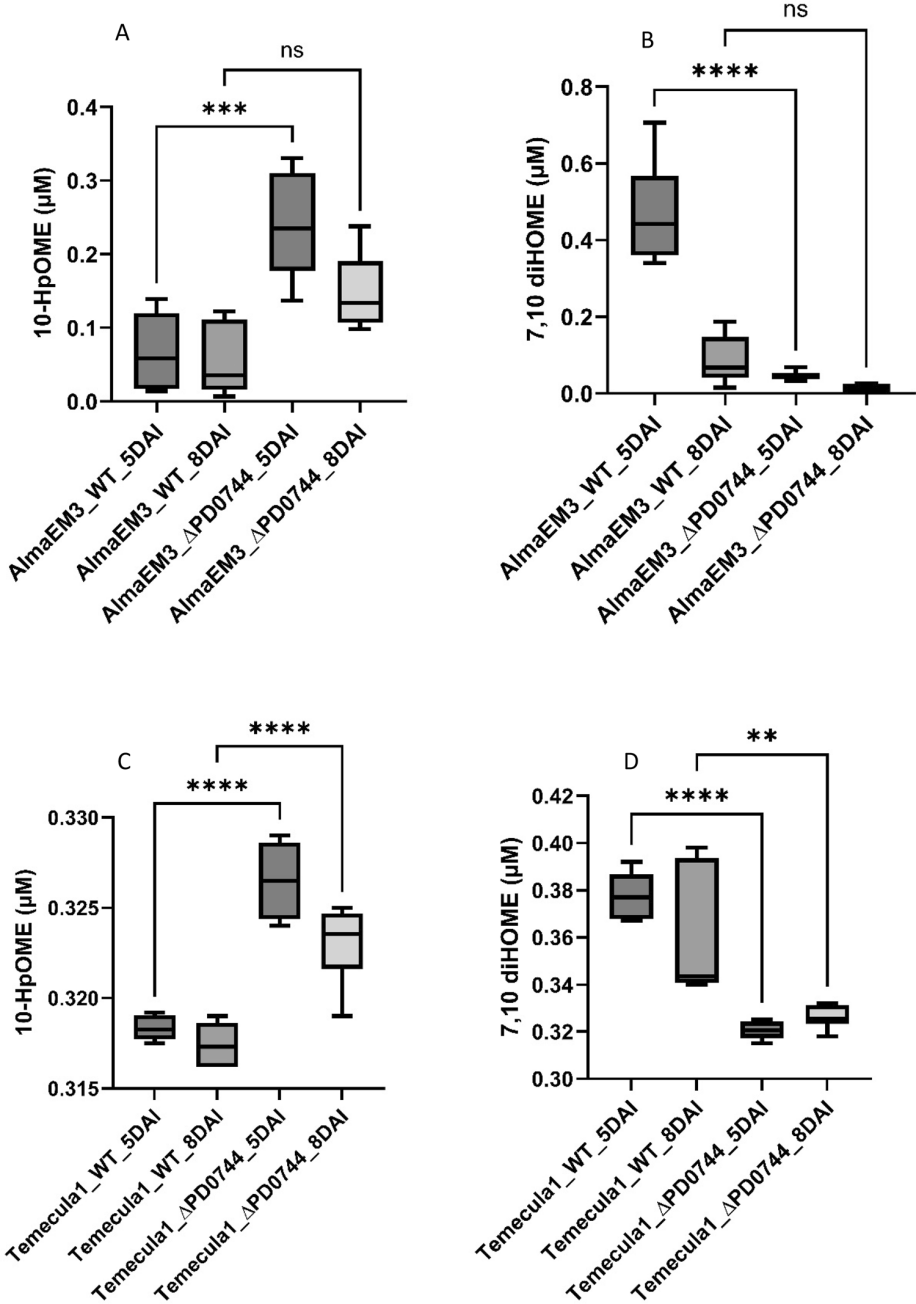
Quantification of oxylipins (10-HpOME and 7,10di-HOME) in wild type (WT) and PD0744 mutant (*ΔPD0744*) of *X. fastidiosa* subsp. *multiplex* strain AlmaEm3 (**A** and **B**) and subsp. *fastidiosa* strain Temecula1 (**C** and **D**) cell extract. Axis y: Concentration of X-axis: Samples from 5-day and 8-day after-inoculation (DAI) mutant and wild type cells. Boxplots were plotted using Tukey method, values were compared by an ordinary one-way ANOVA followed by the Tukey’ post hoc test. Asterisks indicate p value: **** p < 0.0001; ***p < 0.001; **p < 0.01; *p < 0.05)

The oxylipin extraction was carried out on cells of *Xylella fastidiosa* Temecula1 and AlmaEM3 wild type strains and their ∆PD0744 knockout mutants. Cells were grown in PD3 medium for 5 and 8 days, which is the time interval in which Temecula1 and Alma EM3 produce biofilm in liquid culture. 10-HpOME quantification from cell extracts showed an significant increase in AlmaEM3_∆PD0744 mutant strain at 5 dai, but not at 8 dai (Fig. 4A), and the same trend was described for Temecula1_ ∆PD0744 (Fig. 4C), significantly increased both at 5 and 8 dai. Conversely, the 7,10-diHOME oxylipin showed a significant decrease in AlmaEM3_∆PD0744 (Fig. 4B) at 5 dai, and the same trend was represented in Temecula1_ ∆PD0744 (Fig. 4D) mutants both at 5 and 8 dai. The mutation may therefore have limited the metabolic pathway leading to 7,10-DiHOME while promoting an accumulation of its precursor 10-HpOME. This trend is maintained, even if with differences in absolute amount, in both strains.

## Discussion

Results from previous studies show that biofilm formation in *X. fastidiosa* is a complex process involving the contribution of both nonfimbrial and fimbrial adhesins; in fact, disruption of either non-fimbrial or fimbrial adhesins was sufficient to greatly reduce the formation of biofilm rings in culture flasks [4]. Knockout mutants of *X. fastidiosa* subsp. *fastidiosa* strain Temecula1 for the haemagglutinins genes *hxfA* or *hxfB*, show a loss of microcolony and biofilm formation in vitro and *in planta* and a decrease in the affinity to polysaccharides found in the insects’ foregut [10]. In Temecula1 strain, XadA1 mutants were reported to be defective in adhesion to surfaces, while cell–cell aggregation is not affected [3]. If non-fimbrial adhesins seem to be mainly involved in surface adhesion, fimbrial adhesins are mainly involved in cell–cell aggregation as suggested by the fact that FimA– and FimF– mutants did not form aggregates in vitro [3]. These genes may not be expressed in planktonic cells, as they typically do not self-aggregate in liquid cultures [3]. However, they might be expressed when cell density increases and in the biofilm formation process. In this context, the diffusible signal factor (DSF), or other autoinducer molecules like oxylipins, could trigger the expression of signaling-dependent genes. Conversely, it could be expected that the non-fimbrial adhesins, such as the XadA family and the hemagglutinins, that seem to have a prominent role in surface attachment and not in cell–cell aggregation [3, 8, 14], might be expressed even in planktonic cells, thus allowing the adherence of such cells to surfaces as soon as they find them.

In this study we focused on the PD0744 gene product: XadA2, a predicted trimeric autotransporter adhesin. As an non-fimbrial adhesin, it may have a role in the first phase of sessile transition, in the attachment to xylem surface, as suggested by the high affinity for insect chitin and xylem cellulose demonstrated for 9a5c strain in vitro [14], even if previous studies suggested, conversely, that it could be synergistic to fimbrial adhesins in cell–cell interaction [13]. Two knockout mutants for the PD0744 gene, named Xf∆PD0744, were obtained applying a protocol for gene deletion that exploits the natural competence of *X. fastidiosa* subsp. *fastidiosa* Temecula1 and subsp. *multiplex* AlmaEm3 cells [25]. Several tests were conducted to highlight phenotypic differences between the mutant strain and the wild strain. Assays for the evaluation of planktonic growth and biofilm development, carried out in 96-well microplates, showed significant differences between the 2 examined strains. The planktonic phase seems to prevail in the mutant, while in the wild type strain a greater production of biofilm is observed. This is coherent with previously studies in Temecula1, reporting that the mutants for XadA1, paralog of our gene of interest, were defective in adhesion to surfaces [3], whereas wild type 9a5c strain had high affinity for insect chitin and xylem cellulose in vitro due to XadA2 adhesin [14].

It was demonstrated that *X. fastidiosa* knockout mutants for *rpfF* gene (required for synthesis of DSF) [15], *hxfA* gene [hemagglutinin adhesin gene (PD2118)] [30] and type I pilus genes [4] have increased twitching motility, while knockout mutant for type IV pilus genes (*fimT*, *pilX*, *pilY1*, *pilO*, *pilR, fimA)* [4, 7, 25] have a loss in twitching motility. So far, twitching motility was not tested for Trimeric autotransporter adhesins (TAA) non-fimbrial adhesins mutants. The twitching assay carried out for our mutant highlighted how the mutant lost this motility, since no growth fringe was found outside the inoculum margin. Twitching is a motility requiring the cells to be adhered to a substrate [4]. We hypothesize that the absence of XadA2 does not mediate adhesion to extracellular matrix components, based on findings reported by the authors [14], who demonstrated the role of the non fimbrial adhesin XadA2 in facilitating binding and biofilm formation of X. fastidiosa subsp. pauca strain 9a5c on vector surfaces.

HPLC / MS–MS analyses were conducted on cells and cultural filtrates of both the mutant and the wild type strains, both grown in PD3 liquid culture in order to detect the presence of lipid species of interest involved in quorum sensing and pathogen-plant interaction as demonstrated by several authors [6, 16, 18, 31, 32]. In recent years, several studies have highlighted the role of the lipid component in plant pathogen interaction [33, 33, 34] and their role in bacterial cell signalling [21]. We were interested in evaluating the oxylipin profile of the mutant, with particular regard to oxylipins involved in ODS quorum sensing described in *P. aeruginosa i.e.* 10-HpOME (10-hydroperoxyoctadecamonoenic acid) and 7,10-DiHOME (7,10-dihydroxyoctadecamonoenic acid) [21] and recently found in *Nicotiana tabacum* plants artificially inoculated with *X. fastidiosa* subsp*. pauca* strain De Donno [35]. According to mass spectrometry analysis performed in this work, our mutants showed an increase of 10HpOME and a decrease of 7.10-DiHOME (Fig. 4). As a result, the depletion of the XadA2 adhesin may have blocked the 7,10-DiHOME biosynthesis pathway thus leading to an accumulation of its precursor 10 HpOME. In the light of the above data, obtained through the HPLC–MS analyses and aforementioned assays, the mutant cells produced less 7,10-DiHOME compared to the wild type, formed less biofilm in the microplate wells and did not move via twitching. Biofilm formation and twitching motility are essential for the virulence of the bacterium: They both presuppose adhesion to the surface of the xylem vessels [36] and the 7.10-DiHOME oxylipin could be a signal molecule that induces cellular adhesion of the bacterium in vitro*.* Interestingly, according to previous study [37], in vitro growth assays performed in liquid culture revealed that 9-HODE (9 hydroxyoctadecadienoic acid), an oxylipin derived from lipoxygenase (LOX) activity, promoted biofilm formation, while dioxygenase (DOX) -derived oxylipins, 7.10-DiHOME and 10-HpOME have strongly inhibited it [37]. However, concentrations used in that study were much higher (from 8 to 80 µM) than concentrations detected by HPLC/MS–MS in this work (~ 0.4 µM). Thus, it could be hypothesized that at high concentration the 7,10-DiHOME promotes planktonic growth, which allows the systemic spread of the bacterium, and inhibits the formation of biofilm. A dose–response relationship, with a promoter effect of adhesion at low concentrations and an inhibitor effect on biofilm at high concentrations, is therefore not to be excluded. From the data obtained, it could be hypothesized that the absence of XadA2 disrupts the balance between adhesive and non-adhesive cellular phenotypes. We hypothesize that, in maintaining cellular homeostasis, the bacterium may inhibit the accumulation of oxylipins like 7,10-DiHOME, which could otherwise destabilize the switch between non-adhesive and adhesive states.

Adhesins such as XadA2 seem necessary for a first adhesion to the substrate and only later there would be the action of fimbrial adhesins, which favour cell–cell adhesion [14]. The expression of fimbrial adhesins is probably regulated not only by DSF signaling molecules but also by other molecules such as: 7.10-DiHOME and 10-HpOME could be the candidates. The accumulation of their precursor 10-HpOME in the mutants could be the result of the inhibition of the enzyme responsible for its synthesis. We hypothesize that the XadA2 could be involved in the synthesis of 10-HpOME and 7.10-DiHOME, thus the deletion of this gene and consequently the incomplete adhesion process, also affects the biosynthesis of oxylipin.

Moreover, we speculate that the metabolic pathway, which leads to the synthesis of 10-HpOME and 7.10-DiHOME could therefore be functional to the next phase. In other words, the products of this biosynthetic pathway would act as signal molecules capable of favouring the expression of fimbrial adhesins, maybe acting as possible auxiliary signals to the DSF. The entire process leading to biofilm formation and twitching motility, both depending on fimbrial adhesins, after a prior required surface attachment, would therefore be the result of a complex concertation of different and redundant signals.

## Conclusion

We can speculate the presence of an autoinducer loop that involves different signals to modulate the adhesion and biofilm process. Future studies should investigate the possible existence of a quorum sensing operon in this species that can be switched on not only by “autocrine” oxylipin signals but even by paracrine signals produced, for instance, by the infected host as suggested by our previous results. Deciphering this network of signals can pave the way for a tailored control of the lifestyle switches in *Xylella fastidiosa:* Using a specific oxylipin at a specific concentration could switch off the passage from planktonic growth to biofilm formation, limiting essentially the ability of this pathogen to cause xylem blockage and plant disease.

In the future, the ability of WT and mutant strains generated in this study to cause symptoms and colonize the xylem of plants will be compared. Experiments will initially be conducted on tobacco as a model system, followed by grapevines and blueberries as natural hosts of the Temecula and AlmaEm3 strains, respectively. It is anticipated that the PD0744 mutants will have impaired effects on plants, particularly they will be unable to colonize host plants basipetally due to a lack of twitching motility. Moreover, it can be speculated that the mutants will cause less severe symptoms as they produce less biofilm. Experiments using microfluidic chambers will assess the ability of these strains to form biofilm and move under flow conditions, and it is expected that they will exhibit the same phenotypes mentioned above.

## Supplementary Information

Below is the link to the electronic supplementary material.

Supplementary file1 (PDF 48 KB)

## Data Availability

No datasets were generated or analysed during the current study.

## Abbreviations

7,10-DiHOME: 7,10-Dihydroxy-Octamonoenoic Acid
10-HpOME: 10-Hydroperoxy- Octamonoenoic Acid
ODS: Oxylipin-Dependent Quorum Sensing
DSF: Diffusible Signalling Factors

## References

[1] Roper C, Castro C, Ingel B (2019) Xylella fastidiosa: bacterial parasitism with hallmarks of commensalism. Curr Opin Plant Biol 50:140–147 31229798 10.1016/j.pbi.2019.05.005

[2] Kung SH, Almeida RPP (2014) Biological and genetic factors regulating natural competence in a bacterial plant pathogen. Microbiology 160:37–46 24149707 10.1099/mic.0.070581-0

[3] Feil H, Feil WS, Lindow SE (2007) Contribution of fimbrial and afimbrial adhesins of Xylella fastidiosa to attachment to surfaces and virulence to grape. Phytopathology 97:318–324 18943651 10.1094/PHYTO-97-3-0318

[4] Li Y, Hao G, Galvani CD et al (2007) Type I and type IV pili of Xylella fastidiosa affect twitching motility, biofilm formation and cell–cell aggregation. Microbiology 153:719–726 17322192 10.1099/mic.0.2006/002311-0

[5] Meng Y, Li Y, Galvani CD et al (2005) Upstream migration of Xylella fastidiosa via pilus-driven twitching motility. J Bacteriol 187:5560–5567 16077100 10.1128/JB.187.16.5560-5567.2005 PMC1196070

[6] Chatterjee S, Almeida RPP, Lindow S (2008) Living in two worlds: the plant and insect lifestyles of Xylella fastidiosa. Annu Rev Phytopathol 46:243–271 18422428 10.1146/annurev.phyto.45.062806.094342

[7] Merfa MV, Zhu X, Shantharaj D et al (2023) Complete functional analysis of type IV pilus components of a reemergent plant pathogen reveals neofunctionalization of paralog genes. PLoS Pathog 19:e1011154 36780566 10.1371/journal.ppat.1011154 PMC9956873

[8] Rojas CM, Ham JH, Deng WL et al (2002) HecA, a member of a class of adhesins produced by diverse pathogenic bacteria, contributes to the attachment, aggregation, epidermal cell killing, and virulence phenotypes of Erwinia chrysanthemi EC16 on Nicotiana clevelandii seedlings. Proc Natl Acad Sci 99:13142–13147 12271135 10.1073/pnas.202358699 PMC130600

[9] Feitosa-Junior OR, Stefanello E, Zaini PA et al (2019) Proteomic and metabolomic analyses of Xylella fastidiosa OMV-enriched fractions reveal association with virulence factors and signaling molecules of the DSF family. Phytopathology 109:1344–1353 30973310 10.1094/PHYTO-03-19-0083-R

[10] Voegel TM, Warren JG, Matsumoto A et al (2010) Localization and characterization of Xylella fastidiosa haemagglutinin adhesins. Microbiology 156:2172–2179 20378647 10.1099/mic.0.037564-0

[11] Mühlenkamp M, Oberhettinger P, Leo JC et al (2015) Yersinia adhesin A (YadA)–beauty & beast. Int J Med Microbiol 305:252–258 25604505 10.1016/j.ijmm.2014.12.008

[12] Ray SK, Rajeshwari R, Sharma Y, Sonti RV (2002) A high-molecular-weight outer membrane protein of Xanthomonas oryzae pv. oryzae exhibits similarity to non-fimbrial adhesins of animal pathogenic bacteria and is required for optimum virulence. Mol Microbiol 46:637–647 12410822 10.1046/j.1365-2958.2002.03188.x

[13] Caserta R, Takita MA, Targon ML et al (2010) Expression of Xylella fastidiosa fimbrial and afimbrial proteins during biofilm formation. Appl Environ Microbiol 76:4250–4259 20472735 10.1128/AEM.02114-09 PMC2897468

[14] Bossi Esteves M, Lopes Nalin J, Kudlawiec K, Caserta Salviatto R, de Melo ST, Sicard A, Paes P, de Almeida R, Alves de Souza A, Lopes RS (2020) XadA2 adhesin decreases biofilm formation and transmission of Xylella fastidiosa subsp. pauca. Insects 11(8):473 32722654 10.3390/insects11080473 PMC7469142

[15] Chatterjee S, Wistrom C, Lindow SE (2008) A cell–cell signaling sensor is required for virulence and insect transmission of Xylella fastidiosa. Proc Natl Acad Sci 105:2670–2675 18268318 10.1073/pnas.0712236105 PMC2268194

[16] Ionescu M, Yokota K, Antonova E et al (2016) Promiscuous diffusible signal factor production and responsiveness of the Xylella fastidiosa Rpf system. MBio 7:e01054-e1116 27435463 10.1128/mBio.01054-16 PMC4958263

[17] Lindow S, Newman K, Chatterjee S et al (2014) Production of Xylella fastidiosa diffusible signal factor in transgenic grape causes pathogen confusion and reduction in severity of Pierce’s disease. Mol Plant-Microbe Interact 27:244–254 24499029 10.1094/MPMI-07-13-0197-FI

[18] Beaulieu ED, Ionescu M, Chatterjee S, Yokota K, Trauner D, Lindow S (2013) Characterization of a diffusible signaling factor from Xylella fastidiosa. MBio 4(1):10–128 10.1128/mBio.00539-12 PMC3546559 23300249

[19] Martínez E, Cosnahan RK, Wu M et al (2019) Oxylipins mediate cell-to-cell communication in Pseudomonas aeruginosa. Commun Biol 2:1–10 30793044 10.1038/s42003-019-0310-0 PMC6377657

[20] Pohl CH, Kock JLF (2014) Oxidized fatty acids as inter-kingdom signaling molecules. Molecules 19:1273–1285 24448067 10.3390/molecules19011273 PMC6270766

[21] Martinez E, Campos-Gomez J (2016) Oxylipins produced by *Pseudomonas aeruginosa* promote biofilm formation and virulence. Nat Commun 7:13823. 10.1038/ncomms13823 27929111 10.1038/ncomms13823 PMC5155153

[22] Martínez E, Campos-Gómez J (2016) Oxylipins produced by Pseudomonas aeruginosa promote biofilm formation and virulence. Nat Commun 7:13823 27929111 10.1038/ncomms13823 PMC5155153

[23] Battilani P, Lanubile A, Scala V et al (2018) Oxylipins from both pathogen and host antagonize jasmonic acid-mediated defence via the 9-lipoxygenase pathway in Fusarium verticillioides infection of maize. Mol Plant Pathol 19:2162–2176 29660236 10.1111/mpp.12690 PMC6638020

[24] Scala V, Salustri M, Loreti S, Pucci N, Cacciotti A, Tatulli G, Scortichini M, Reverberi M (2022) Mass spectrometry-based targeted lipidomics and supervised machine learning algorithms in detecting disease, cultivar, and treatment biomarkers in Xylella fastidiosa subsp pauca-infected olive trees. Front Plant Sci 13:833245 35528940 10.3389/fpls.2022.833245 PMC9072861

[25] Kandel PP, Chen H, De La Fuente L (2018) A short protocol for gene knockout and complementation in Xylella fastidiosa shows that one of the type IV pilin paralogs (PD1926) is needed for twitching while another (PD1924) affects pilus number and location. Appl Environ Microbiol 84:e01167-e1218 29980551 10.1128/AEM.01167-18 PMC6121978

[26] Vieira J, Messing J (1982) The pUC plasmids, an M13mp7-derived system for insertion mutagenesis and sequencing with synthetic universal primers. Gene 19:259–268 6295879 10.1016/0378-1119(82)90015-4

[27] Cruz LF, Cobine PA, De La Fuente L (2012) Calcium increases Xylella fastidiosa surface attachment, biofilm formation, and twitching motility. Appl Environ Microbiol 78:1321–1331 22194297 10.1128/AEM.06501-11 PMC3294462

[28] Scala V, Reverberi M, Salustri M et al (2018) Lipid profile of Xylella fastidiosa Subsp. pauca associated with the olive quick decline syndrome. Front Microbiol 9:1839 30154768 10.3389/fmicb.2018.01839 PMC6102392

[29] Galvani CD, Li Y, Burr TJ, Hoch HC (2007) Twitching motility among pathogenic Xylella fastidiosa isolates and the influence of bovine serum albumin on twitching-dependent colony fringe morphology. FEMS Microbiol Lett 268:202–208 17328746 10.1111/j.1574-6968.2006.00601.x

[30] Guilhabert MR, Kirkpatrick BC (2005) Identification of Xylella fastidiosa antivirulence genes: hemagglutinin adhesins contribute to X. fastidiosa biofilm maturation and colonization and attenuate virulence. Mol Plant-Microbe Interact 18:856–868 16134898 10.1094/MPMI-18-0856

[31] Von Bodman SB, Bauer WD, Coplin DL (2003) Quorum sensing in plant-pathogenic bacteria. Annu Rev Phytopathol 41:455–482 12730390 10.1146/annurev.phyto.41.052002.095652

[32] He Y-W, Zhang L-H (2008) Quorum sensing and virulence regulation in Xanthomonas campestris. FEMS Microbiol Rev 32:842–857 18557946 10.1111/j.1574-6976.2008.00120.x

[33] Van Meer G, Voelker DR, Feigenson GW (2008) Membrane lipids: where they are and how they behave. Nat Rev Mol cell Biol 9:112–124 18216768 10.1038/nrm2330 PMC2642958

[34] Sohlenkamp C, Geiger O (2016) Bacterial membrane lipids: diversity in structures and pathways. FEMS Microbiol Rev 40:133–159 25862689 10.1093/femsre/fuv008

[35] Siebers M, Brands M, Wewer V et al (2016) Lipids in plant–microbe interactions. Biochim Biophys Acta Mol Cell Biol Lipids 1861:1379–1395. 10.1016/j.bbalip.2016.02.021 10.1016/j.bbalip.2016.02.021 26928590

[36] Cruz LF, Parker JK, Cobine PA, De La Fuente L (2014) Calcium-enhanced twitching motility in Xylella fastidiosa is linked to a single PilY1 homolog. Appl Environ Microbiol 80:7176–7185 25217013 10.1128/AEM.02153-14 PMC4249194

[37] Scala V, Pucci N, Salustri M et al (2020) Xylella fastidiosa subsp. pauca and olive produced lipids moderate the switch adhesive versus non-adhesive state and viceversa. PLoS ONE 15:e0233013 32413086 10.1371/journal.pone.0233013 PMC7228078

