# Supplementary material for: XadA-like adhesin XADA2 regulates biofilm formation in X. fastidiosa subsp. fastidiosa putatively by engaging oleic-acid derived oxylipins
Source: Mol Biol Rep. 2025 Feb 25;52(1):263. doi: 10.1007/s11033-025-10259-y (PMC11861136; doi:10.1007/s11033-025-10259-y)

Fig S1. Amplicons of upstream region (UP), downstream region (DOWN), kanamycin resistance cassette (KAN), mutagenesis construct (FUS), internal control region (INT). As evident from line 7 to 10, relative to the deletion confirmation, the knockout mutant (lanes 9 and 10) lacks the INT amplicon while having the KAN, whilst for the wild type strain (lanes 7 and 8) the situation is the opposite.

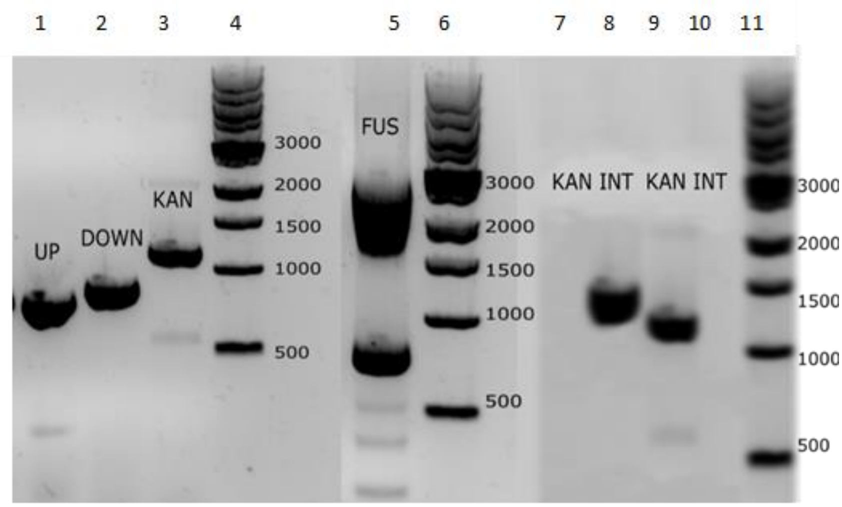

Supplement: Supplementary file 1 — Supplementary file1 (PDF 48 KB) [file 11033_2025_10259_MOESM1_ESM.pdf]
